# AICAR Ameliorates Non-Alcoholic Fatty Liver Disease via Modulation of the HGF/NF-κB/SNARK Signaling Pathway and Restores Mitochondrial and Endoplasmic Reticular Impairments in High-Fat Diet-Fed Rats

**DOI:** 10.3390/ijms24043367

**Published:** 2023-02-08

**Authors:** Doaa Hussein Zineldeen, Nahid Mohamed Tahoon, Naglaa Ibrahim Sarhan

**Affiliations:** 1 Medical Biochemistry and Molecular Biology Department, Faculty of Medicine, Tanta University, Tanta 6632110, Egypt; 2 College of Medicine, Sulaiman AlRajhi University, Albukairiyah 51942, Saudi Arabia; 3 Physiology Department, Faculty of Medicine, Tanta University, Tanta 6632110, Egypt; 4 Histology Department, Faculty of Medicine, Tanta University, Tanta 6632110, Egypt

**Keywords:** NAFLD, AICAR, HGF, NF-κB, SNARK, SIRT2, mitochondria, ER stress

## Abstract

Non-alcoholic fatty liver disease (NAFLD) is a global health problem characterized by altered lipid and redox homeostasis, mitochondrial dysfunction, and endoplasmic reticulum (ER) stress. The AMP-dependent kinase (AMPK) agonist 5-aminoimidazole-4-carboxamide ribonucleoside (AICAR) has been shown to improve the outcome of NAFLD in the context of AMPK activation, yet the underlying molecular mechanism remains obscure. This study investigated the potential mechanism(s) of AICAR to attenuate NAFLD by exploring AICAR’s effects on the HGF/NF-κB/SNARK axis and downstream effectors as well as mitochondrial and ER derangements. High-fat diet (HFD)-fed male Wistar rats were given intraperitoneal AICAR at 0.7 mg/g body weight or left untreated for 8 weeks. In vitro steatosis was also examined. ELISA, Western blotting, immunohistochemistry and RT-PCR were used to explore AICAR’s effects. NAFLD was confirmed by steatosis score, dyslipidemia, altered glycemic, and redox status. HGF/NF-κB/SNARK was downregulated in HFD-fed rats receiving AICAR with improved hepatic steatosis and reduced inflammatory cytokines and oxidative stress. Aside from AMPK dominance, AICAR improved hepatic fatty acid oxidation and alleviated the ER stress response. In addition, it restored mitochondrial homeostasis by modulating Sirtuin 2 and mitochondrial quality gene expression. Our results provide a new mechanistic insight into the prophylactic role of AICAR in the prevention of NAFLD and its complications.

## 1. Introduction

Non-alcoholic fatty liver disease (NAFLD) is a global chronic metabolic disorder characterized by triacylglycerol (TAG) accumulation in the liver with minute or no alcohol consumption; it is commonly associated with metabolic risk factors, including obesity, dyslipidemia, insulin resistance, and diabetes mellitus [1,2]. NAFLD is highly prevalent worldwide, with higher rates in the Middle East (32%) [1,3,4]. NAFLD patients are at heightened risk of developing NASH (non-alcoholic steatohepatitis), fibrosis, cirrhosis, and hepatocellular carcinoma [5]. Therefore, there is an urge to identify therapeutic targets and drugs to protect against NAFLD [1]. NAFLD’s molecular pathogenesis is better explained by the multiple hit theory involving mitochondrial dysfunction, reactive oxygen species (ROS) generation, oxidative stress, endoplasmic reticulum (ER) stress and immunoinflammatory responses [1].

Mitochondria play an essential role in oxidation and cellular metabolism. Mitochondrial morphology affects its function; mitochondria exist as a dynamic network and undergo constant balanced fission and fusion events [6]. Fusion promotes complementation among damaged mitochondria to overcome cellular stress. Fission is a process that leads to the creation of multiple new mitochondria, and it is primarily regulated by a single GTPase dynamin-related protein 1 (Drp1) [6]. Hepatic mitochondrial fission/fusion dynamics are altered in NAFLD, i.e., increased fission, which plays a central role in NAFLD pathogenesis [1,7]. Liver biopsies from NAFLD patients reveal structural mitochondrial fragmentation, cristolysis, and the formation of giant mitochondria [8]. In vivo and in vitro steatosis models exhibit fragmented mitochondria with heightened Drp1-mediated fission machinery [7,9]. Mitochondrial dysfunction results mainly from oxidative stress, with abnormalities in the mitochondrial electron transport chain (ETC) and oxidative phosphorylation. However, the exact mechanism of hepatic mitochondrial dysfunction in NAFLD remains largely elusive [1].

One proposed mechanism of mitochondrial dysfunction is the dysregulation of the precise signaling between the ER and mitochondria via a calcium ion imbalance; this can be seen in multiple metabolic and neurological diseases [10]. For example, clinical and experimental studies have reported the involvement of ER stress signaling in steatosis and steatohepatitis [11]. Lipotoxic stress causes the ER to activate an adaptive program, known as the unfolded protein response (UPR), to reestablish equilibrium. Initiation of UPR engages three different signaling pathways, each mediated by protein kinase-R-like ER kinase (PERK), inositol requiring enzyme-1α (IRE-1α), and activating-transcription factor-6 (ATF6), respectively. The combined action of the PERK/IRE-1α/ATF6 pathways leads to either recovery from ER stress or progression to hepatic steatosis and steatohepatitis. Targeting these pathways has been proven to halt NAFLD progression [2,11,12].

Hepatic ER stress and UPR induction of the PERK/IRE-1α/ATF6 pathways induce oxidative stress, generates ROS, and promotes hepatic fatty changes [12]. These in turn trigger a variety of stress-sensitive intracellular signaling pathways, primarily NF-κB. Meanwhile, circulating cytokines and exerkines released by the skeletal muscles and adipose tissue of obese individuals are implicated in the immunometabolic derangements seen in NAFLD [13]. In this regard, lipid peroxidation products bind to hepatocyte proteins and initiate an immune response, neutrophil infiltration, and steatohepatitis, which lead to NF-κB activation in the liver [14]. NF-κB activation stimulates the transcription of hepatocyte growth factor (HGF), which is a hepatocyte mitogen elevated in sera of patients with NAFLD, obesity, hypertension, and metabolic syndrome [15,16,17]. HGF signaling is initiated by binding to its tyrosine kinase receptor (c-MET), followed by the phosphorylation and activation of downstream effectors such as phosphoinositide 3 kinase (PI3K), signal transducer and activator of transcription 3 (STAT3), mitogen-activated protein kinases (MAPK), and extracellular signal-regulated protein kinase (ERK). These trigger the activation of NF-κB and hypoxia-inducible factor 1-α (HIF-1α) [18,19]. One of the NF-κB transcriptional targets is sucrose non-fermenting AMPK-related kinase (SNARK). SNARK is a member of the AMP-dependent kinase family of serine/threonine kinases and acts as a stress-response transcription modulator. SNARK is the only kinase from this family that is induced by TNF-α in response to NF-κB activation [20]. It is expressed in a subset of altered metabolic conditions, such as palmitate accumulation, impaired glucose metabolism of skeletal muscles in obese individuals, and obesity in animals fed a high-fat diet (HFD) [21].

To prevent and treat NAFLD, different drugs are currently used (or are in clinical trials) to alleviate hepatic mitochondrial dysfunction. Drugs that increase cellular NAD^+^ and activate Sirtuin 2 (SIRT2), an NAD^+^-dependent deacetylase, have improved mitochondrial dysfunction [2,22]. The AMP-activated protein kinase (AMPK) activators such as metformin are widely used in NAFLD management, but the exact mechanism of action remains largely ill-defined [1]. AICAR possesses anti-inflammatory and antioxidant properties that enhance mitochondrial function due to its ability to stimulate mitochondrial biogenesis without altering the mitochondrial membrane potential and to decrease ROS generation [23]. However, the underlying molecular mechanism for how AICAR prevents mitochondrial dysfunction remains to be identified [24]. Mechanistically, AICAR is converted inside the cell into 5-aminoimidazole-4-carboxamide-1-β-D-ribofuranosyl monophosphate (ZMP), a metabolite in the de novo purine synthesis pathway, which mimics AMP and activates AMPK irrespective of cellular energy status [25]. Meanwhile, AICAR also has multiple AMPK-independent effects, such as control of carbohydrate metabolism, hepatic oxidative phosphorylation, and restriction of cell cycle progression. These AICAR-specific effects could be attributed to its direct ability to modulate transcription machinery, so it may be more favorable than other AMPK agonists [24,25,26].

In the present study, we sought to investigate the underlying molecular mechanisms that govern how AICAR mitigates NAFLD aside from AMPK dependence. The modulatory effects of AICAR on the HGF/NF-κB signaling pathway and its downstream effectors were analyzed. The impact of AICAR on ER stress, mitochondrial dysfunction, and redox status was also investigated. Our findings challenge the paradigm that AMPK is mandatory for AICAR to be protective against steatosis.

## 2. Results

### 2.1. AICAR Attenuates Body Weight Changes and Hepatic Steatosis in HFD-Induced NAFLD Rats

**Figure 1:**
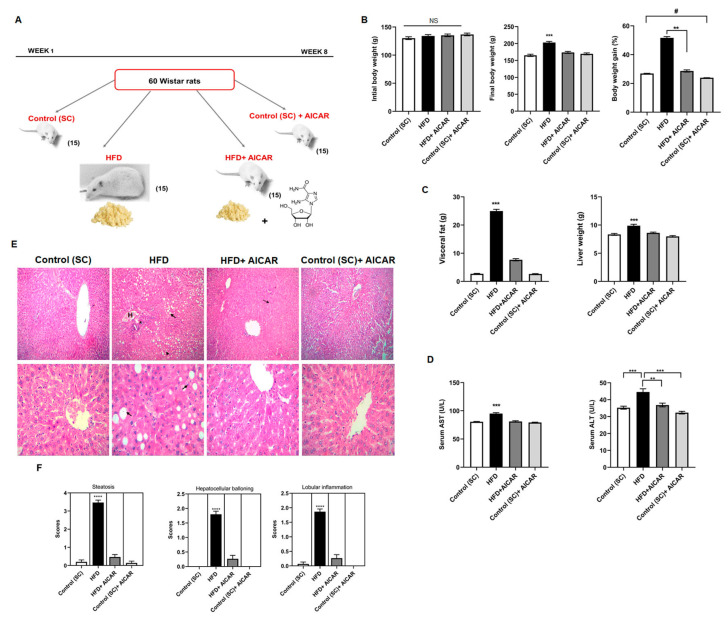
AICAR attenuates body weight changes and protects against hepatic steatosis. (**A**) outline of the experimental groups and treatment used in the study. Male Wistar rats fed on HFD with or without AICAR for 8 weeks. (**B**) Initial body weight, final body weight, and percentage of body weight gain of all groups under study; (***) *p* < 0.001 for HFD vs. all groups; (**) *p* = 0.0061 for HFD vs. HFD + AICAR; (#) *p* = 0.0444 for Control (SC) + AICAR vs. Control (SC). (**C**) Visceral fat weight and liver weight, (***) *p* < 0.001. (**D**) Serum AST and serum ALT, respectively, of all the studied groups in (**A**); (***) *p* < 0.001 for HFD vs. all groups; (**) *p* = 0.0004 for HFD vs. HFD + AICAR. (**E**) Photomicrographs of H&E-stained hepatic tissues from all groups as indicated. The two control groups show normal hepatic architecture. The HFD group reveals vacuolar degeneration, macrovesicular steatosis with signet ring appearance (arrows), ballooning of hepatocytes, Mallory hyaline bodies (arrowheads), inflammatory infiltration (*), and Hemorrhage (H). The HFD + AICAR group shows protective effects on liver architecture as evidenced by less steatosis and fewer signet ring cells and ballooned cells (outline arrow). Magnification: upper panel = 200× and lower panel = 400×. (**F**) NAFLD activity score of steatosis, hepatocellular ballooning, and lobular inflammation; (****) *p* < 0.0001 for all. Significance by one-way ANOVA and/or Kruskal–Wallis test followed by Tukey’s and/or Dunn’s post-hoc test, and data are presented as mean ± SEM, *n* = 15. *p* ≤ 0.05 is considered significant; (NS): non-significant.

We have previously described that HFD-fed rats exhibit a significant increase in body weight and abdominal fat accumulation [27]. After eight weeks of AICAR administration (Figure 1A), the HFD + AICAR group showed decreased body and liver weights and visceral fat when compared to the HFD group (Figure 1B,C; *p* < 0.001). Interestingly, the control + AICAR group showed less weight gain compared to the control (SC) group, indicating the role of AICAR in weight reduction under normal non-lipogenic conditions (Figure 1B; *p* = 0.0444). NAFLD was confirmed by liver enzymes, and the HFD group revealed higher serum activities of transaminases ALT and AST compared to the HFD + AICAR group (Figure 1D; *p* < 0.001, *p* = 0.0004, respectively). In order to confirm NAFLD development, histopathological analysis of H&E-stained liver sections revealed that the HFD group developed steatotic changes in the form of vacuolar degeneration, macrovesicular steatosis with signet ring appearance, ballooning of hepatocytes, Mallory hyaline bodies, and inflammatory infiltration. AICAR effectively reduced fat droplet accumulation in the perivenular and the periportal regions, inflammatory cell infiltrates, and microvesicular steatosis (Figure 1E). This was consistent with the significant decrease in the NAFLD activity score of steatosis, hepatocellular ballooning, and lobular inflammation in the HFD + AICAR group (Figure 1F; *p* < 0.0001).

### 2.2. AICAR Mitigates Hyperglycemia, Insulin Resistance, Altered Lipogenesis Induced by HFD

**Figure 2:**
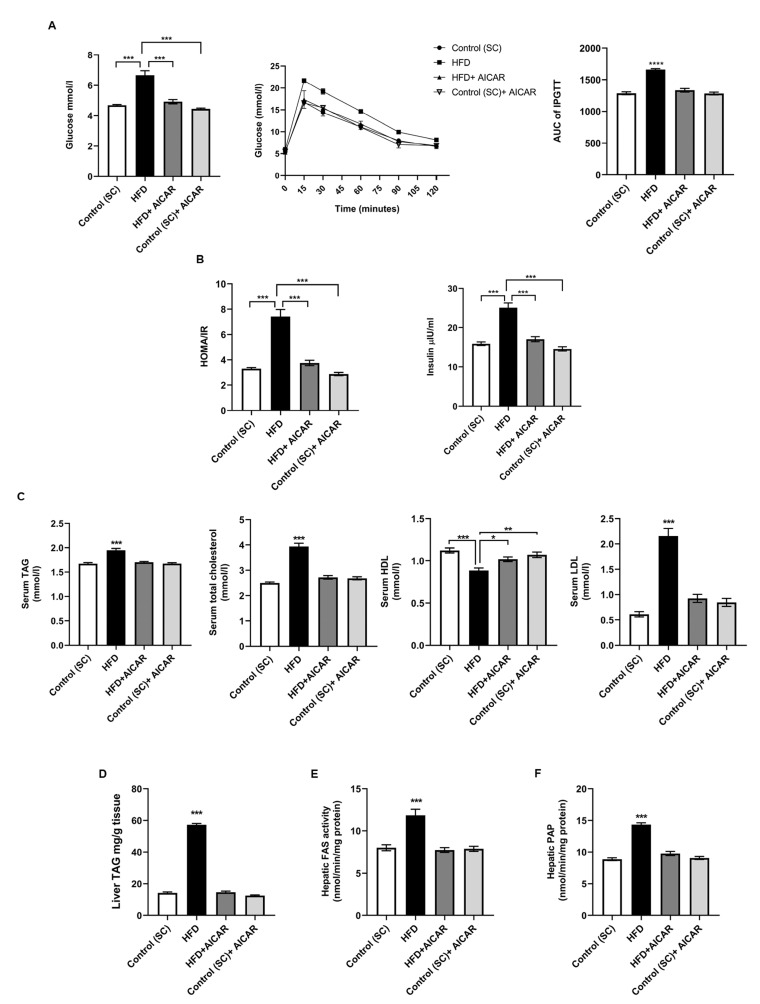
AICAR mitigates hyperglycemia, Insulin resistance and dyslipidemia and normalized hepatic lipogenesis in HFD-fed rats. Eight weeks aged Wistar rats fed on HFD with or without AICAR for 8 weeks. (**A**) Mean FBG, IPGTT at the end of the eighth week, and AUC of IPGTT; (***) *p* < 0.001; (****) *p* < 0.0001 for HFD vs. all groups. (**B**) Insulin and HOMA/IR; (***) *p* < 0.001. (**C**) Serum levels of TAG, cholesterol, HDL, and LDL; (***) *p* < 0.001; (**) *p* = 0.0003; (*) *p* = 0.0160. (**D**–**F**) Liver TAG content, hepatic FAS, and PAP activities, respectively; (***) *p* < 0.001 for HFD vs. all groups. Significance by one-way ANOVA followed by Tukey’s post-hoc test, and data are presented as mean ± SEM, *n* = 15. *p* ≤ 0.05 is considered significant. (FBG: fasting blood glucose; IPGTT: intraperitoneal glucose tolerance test; HOMA/IR: Homeostatic Model Assessment for Insulin Resistance; TAG: triacylglycerol; HDL: high density lipoprotein; LDL: low density lipoprotein; FAS: fatty acid synthase; PAP: phosphatidic acid phosphohydrolase).

NAFLD pathogenesis encompasses altered carbohydrate and lipid metabolism with chronic inflammation [1]. HFD-fed rats had a high fasting blood glucose (FBG) level (*p* < 0.001), impaired intraperitoneal glucose tolerance test (IPGTT) (*p* < 0.0001), fasting insulin, and HOMA-IR index (*p* < 0.001) compared to other groups (Figure 2A,B). Consequently, AICAR significantly reduced FBG, IPGTT and insulin resistance in the HFD + AICAR group. HFD rats displayed an abnormal lipid profile (Figure 2C). On the other hand, HFD + AICAR rats showed lower levels of serum triglycerides (TAG), total and LDL-cholesterol (*p* < 0.001), and serum HDL-cholesterol was increased (*p* = 0.0160 vs. HFD). The regulation of cellular lipid storage plays a critical role in metabolic homeostasis, and its dysregulation may contribute to fatty liver. The lipin/phosphatidic acid phosphatase (PAP) enzyme modulates levels of cellular triacylglycerols [28]. In order to assess the regulatory role of AICAR in hepatic lipogenesis, we assessed hepatic TAG, hepatic fatty acid synthase (FAS), and PAP activities. HFD + AICAR rats exhibited a significant reduction in hepatic triglyceride content as well as FAS activity compared to rats given HFD (Figure 2D,E; *p* < 0.001 for HFD vs. all groups for all). Additionally, AICAR reduced PAP activity in treated rats compared to the HFD group (Figure 2F; *p* < 0.001 for HFD vs. all groups). These results reveal the regulatory role of AICAR on lipid and glucose metabolism in NAFLD rats.

### 2.3. AICAR Administration Inhibits HFD-Induced Steatosis by Modulating the HGF/NF-κB Pathway and Downstream Effectors

**Figure 3:**
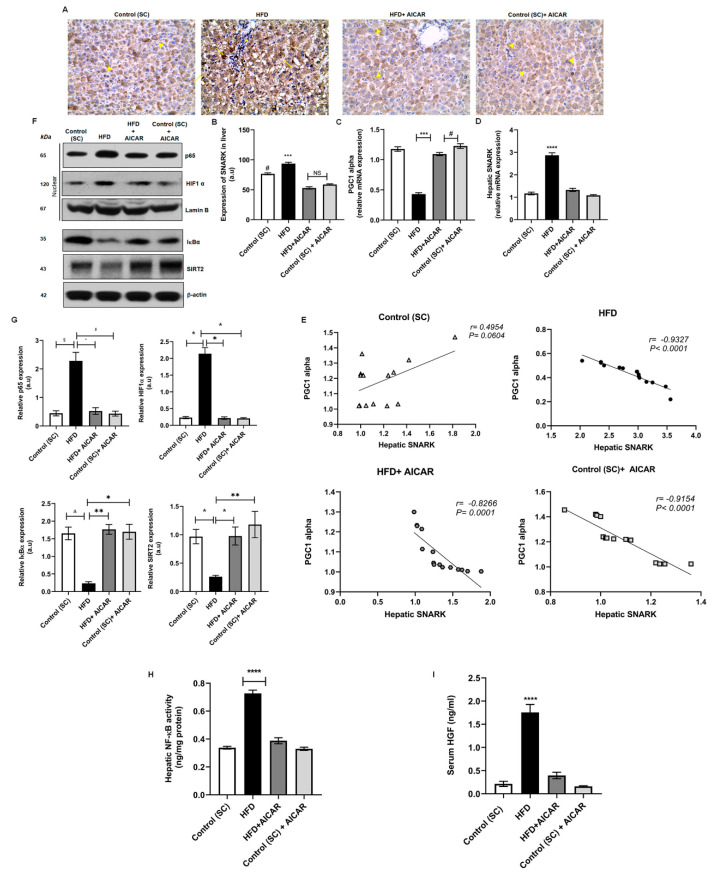
AICAR alleviates HFD-induced steatosis via modulating HGF/NF-κB pathways and downstream effectors. (**A**) Representative photomicrographs of immunohistochemistry staining with SNARK antibodies of hepatic sections from all studied groups as indicated. Arrowhead points at cytoplasmic and membranous staining; arrow points at nuclear staining (X400). (**B**) Densitometry of hepatic SNARK immunoreactivity in all groups in the study; (***) *p* < 0.001 for HFD vs. all groups, (#) *p* < 0.0001 for control vs. all groups (a.u.: arbitrary units). Relative hepatic mRNA expression of (**C**) *PGC1-α* ([***] *p* < 0.001, [#] *p* < 0.05) and (**D**) *SNARK* ([****] *p* < 0.0001) for HFD vs. all groups. (**E**) Pearson correlation of *PGC1-α* and *SNARK* gene expressions in the liver of all studied groups (*n* = 15). (**F**) Effect of AICAR (0.7 mg/g b.w.) on hepatic protein expression of p65, HIF1-α, IκBα, and SIRT2; hepatic homogenates and nuclear fractions were separated by10% SDS-PAGE followed by Western blotting with the specified antibodies; lamin B1 or β-actin were used as a loading control. (**G**) Densitometric analysis of immunoblots of p65: (§) *p* = 0.0184, (″) *p* = 0.0309, ($) *p* = 0.0131; HIF1-α: (*) *p* < 0.05 for all; IκBα: (*) *p* < 0.05, (**) *p* < 0.01, (∆) *p* = 0.025 and SIRT2: (*) *p* < 0.05, (**) *p* < 0.01 using Kruskal–Wallis test followed by Dunn’s post hoc-test (*n* = 6). (**H**) Hepatic NF-κB activity quantified by ELISA; (****) *p* < 0.0001 for HFD vs. all groups (*n* = 15). (**I**) Serum circulating HGF levels quantified by ELISA; (****) *p* < 0.0001 for HFD vs. all groups (*n* = 15). Significance by one-way ANOVA followed by Tukey’s post-hoc test. Data are presented as mean ± SEM, *n* = 15. *p* ≤ 0.05 is considered significant; (NS): non-significant.

Inflammation is the hallmark of steatosis that is transitioning to NASH [3]. AICAR’s anti-inflammatory actions independent of AMPK have been described [29]. Therefore, we investigated liver inflammatory signaling through HGF and NF-κB/p65. HFD-fed rats exhibited significantly elevated serum HGF values compared to other groups. AICAR significantly reduced circulating HGF levels in the HFD + AICAR group (Figure 3I; *p* < 0.0001). HGF stimulates canonical NF-κB DNA binding by driving IκBα degradation [30].

Hepatic nuclear p65 levels were significantly raised in the HFD group. AICAR diminished hepatic NF-κB activity in the HFD + AICAR group, as evidenced by Western blotting and ELISA (Figure 3F–H; *p* = 0.0309; <0.0001 vs. HFD, respectively). AICAR also augmented the hepatic protein expression of the inhibitor IκBα (Figure 3F,G; *p* < 0.001 vs. HFD). We went further to investigate HGF downstream effector HIF1-α, which in turn represses SIRT2 and peroxisome proliferator-activated receptor gamma co-activator-1α (PGC1-α) expression [22]. HIF1-α protein expression was significantly upregulated in the livers of HFD-fed rats. This was associated with a reduced hepatic SIRT2 protein expression as well as reduced hepatic *PGC1-α* mRNA expression. HFD + AICAR rats revealed reduced HIF1-α protein expression with upregulation of SIRT2 protein levels and *PGC1-α* mRNA levels in hepatic tissues (Figure 3C,F,G; *p* < 0.05, *p* < 0.05, *p* < 0.001 vs. HFD, respectively). Furthermore, we inspected AICAR’s impact on hepatic SNARK expression, a downstream target of NF-κB. Immunohistochemical analysis of SNARK in hepatic tissues displayed cytoplasmic and membranous localization in the control groups. Sections from the HFD group revealed significantly higher SNARK immunoreactivity with nuclear localization. The HFD + AICAR group exhibited reduced SNARK immunoreactivity and restoration of cytoplasmic localization. There was no statistical difference among all AICAR-treated groups regarding SNARK immunoreactivity; however, a significant difference was found when compared to the control group (Figure 3A,B; *p* < 0.0001 for all). When we examined AICAR’s effect on *SNARK* gene expression, HFD-fed rats showed upregulation of hepatic *SNARK* mRNA compared to all groups; meanwhile, AICAR administration significantly reduced hepatic *SNARK* mRNA expression by 2.2-fold. The observed decline in hepatic SNARK gene expression correlated negatively with hepatic *PGC1-α* mRNA expression, which was of statistical significance only in the HFD and AICAR treated groups (Figure 3E; *p* < 0.0001).

### 2.4. Effect of AICAR on Hepatic Ultrastructure and Mitochondrial Quality Control

**Figure 4:**
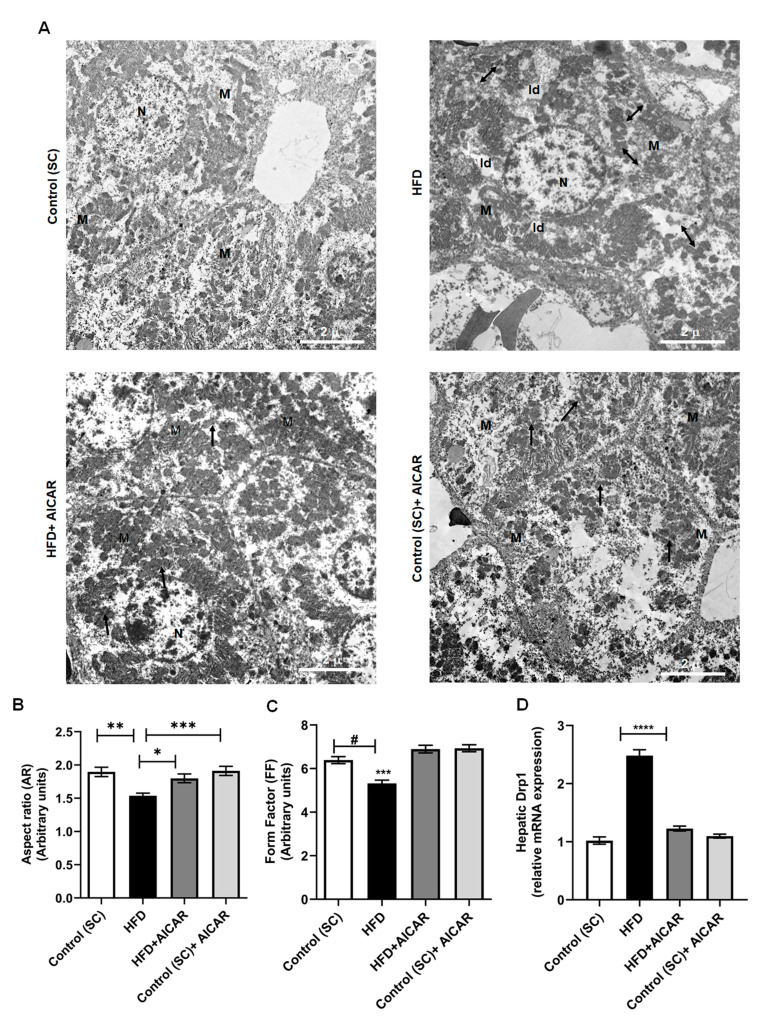
Impact of AICAR treatment on hepatic ultrastructure and mitochondrial morphology. (**A**) Representative photomicrographs of liver tissues from all experimental groups examined under transmission electron microscope as indicated. Control (SC) group showed normal hepatic architecture, hepatocytes are displayed with nucleus (N) and normal-shaped mitochondria (M) with ellipsoidal, tubular, and rounded forms. The HFD group revealed lipid droplets (ld) around the hepatocyte nucleus (N), with patchy areas of clumped mitochondria scattered and dispersed throughout a rarified cytoplasm, (M) dumbbell-shaped mitochondria with fission or fragmentation (double-headed arrow ↔) were also seen. The HFD + AICAR group displayed hepatocytes with a large number of normal-shaped mitochondria (M) distributed throughout the cytoplasm with ongoing fusion (black arrows). The Control (SC) + AICAR group showed normal hepatic and mitochondrial morphology with hepatocytes showing some population in the process of fusion (black arrows). X1000 magnification (scale bar 2 μ). (**B**,**C**) The hepatocyte mitochondrial morphological parameters of aspect ratio (AR) and form factor (FF) were quantified by Fiji Image J software. (*n* = 100) of three independent experiments. Data represented as mean ± SEM by Kruskal–Wallis (K.W) test followed by Dunn’s post-hoc test. (**B**) AR: (K.W: 19.44), (*) *p* < 0.05, (**) *p* < 0.01, (***) *p* < 0.001. (**C**) FF: (K.W: 54.77), (#) *p* = 0.004, (***) *p* < 0.001. (**D**) Relative mRNA expression of the mitochondrial fission protein Dynamin-Related Protein1 (*Drp1*) in liver tissues from the indicated groups. Significance by one-way ANOVA followed by Tukey’s post-hoc test and data are expressed as mean ± SEM, *n* = 15, (F = 4.135), (****) *p* < 0.0001 for HFD vs. other groups.

Fission and fusion act as quality control mechanisms; fusion maintains healthy mitochondria, while fission, followed by mitophagy, gets rid of low-quality mitochondria [6]. Ultrastructural examination of ultrathin sections of livers from HFD-fed animals by transmission electron microscopy showed lipid droplets around the nucleus of hepatocytes, with patchy areas of clumped mitochondria scattered and dispersed throughout a rarified cytoplasm; dumbbell-shaped mitochondria with fission or fragmentation were also seen. Higher magnification revealed degenerative changes, including swelling, rounding up, and loss of cristae in the livers of HFD-fed rats. HFD + AICAR rats displayed fewer lipid droplets and a higher number of normal-shaped mitochondria distributed throughout the cytoplasm with ongoing fusion processes (Figure 4A, Supplementary Figure S1). Interestingly, control animals receiving AICAR also showed an increased number of mitochondria and increased fusion. We then performed a morphometric analysis of hepatic mitochondria by Fiji. We assessed AR (aspect ratio), which reflects mitochondrial length, and FF (form factor), which represents mitochondrial length and degree. AR and FF values increase as mitochondria elongate. Our results revealed that the morphology of mitochondrial hepatocytes was mostly tubular in the control groups. Mitochondria from hepatocytes of the HFD group were considerably truncated and rounded with significantly lower AR and FF values, indicating mitochondrial fragmentation and fission. However, HFD + AICAR treatment prevented mitochondrial fragmentation and maintained normal mitochondrial tubular structure with significantly higher AR and FF values (Figure 4B,C; *p* < 0.05). Dynamin-related protein-1 (Drp1) is an evolutionarily conserved GTPase that promotes mitochondrial fission [6]. We assessed the hepatic expression of *Drp1* as a marker of mitochondrial quality. HFD-fed animals exhibited significantly increased hepatic *Drp1* mRNA when compared to other groups. AICAR downregulated hepatic *Drp1* mRNA expression in the HFD + AICAR group; this is coincident with our electron microscopic finding of decreased mitochondrial fission in the HFD + AICAR and control + AICAR groups. (Figure 4A,D; *p* < 0.0001).

### 2.5. AICAR Alleviates the HFD-Induced Inflammatory Response and Oxidative Stress

**Figure 5:**
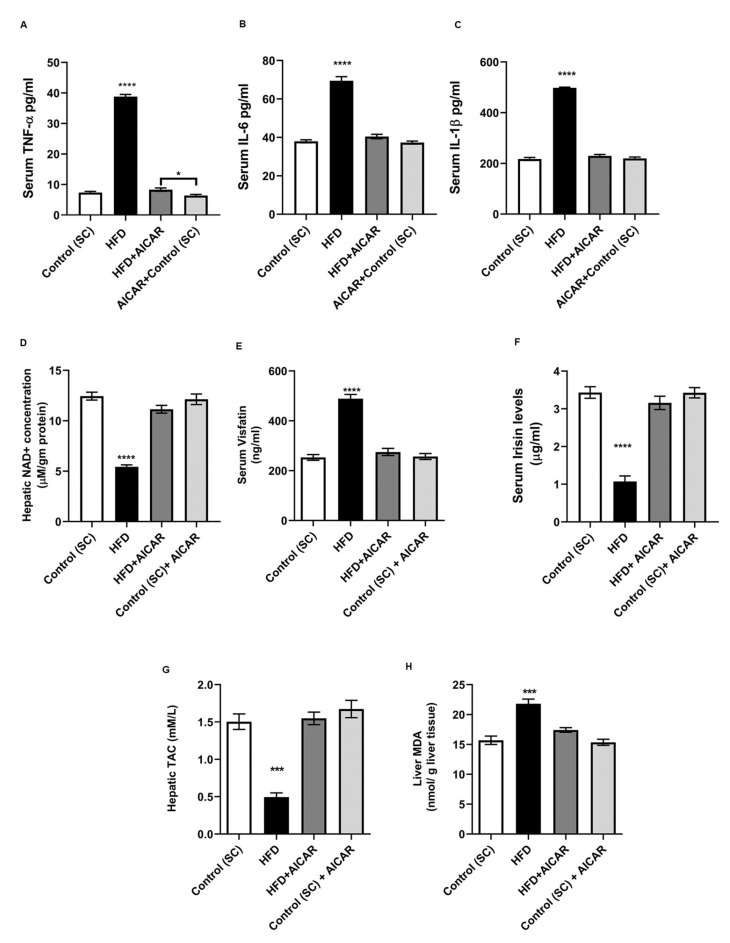
Effects of AICAR on inflammatory cytokines, exerkines, and hepatic redox status in HFD-induced steatosis. Mean circulating levels of inflammatory cytokines assayed by ELISA (**A**) TNF-α: (****) *p* < 0.0001 HFD vs. all groups, (*) *p* = 0.0483 for HFD + AICAR *vs* AICAR control. (**B**,**C**) IL-6 and IL-1β: (****) *p* < 0.0001 for HFD vs. all groups for all. (**D**) Colorimetric assay of hepatic NAD^+^ concentrations among all the studied groups, (****) *p* < 0.0001 for HFD vs. all groups. (**E**,**F**) Mean circulating levels of extracellular visfatin and irisin assayed by ELISA, (****) *p* < 0.0001 for HFD vs. all groups for all. (**G**) Total antioxidant capacity (TAC) of hepatic tissues among the studied groups, (***) *p* < 0.001 for HFD vs. all. (**H**) Liver peroxidation product malondialdehyde (MDA) concentrations of all groups, (***) *p* < 0.001 for HFD vs. all. Significance by one-way ANOVA followed by Tukey’s post-hoc test, and data are presented as mean ± SEM, *n* = 15. *p ≤* 0.05 is considered significant.

Next, we evaluated the effect of AICAR on the HFD-induced inflammatory response and serum proinflammatory cytokines concentrations in NAFLD rats. Circulating TNF-α levels were increased in the HFD group (Figure 5A; *p* < 0.0001 vs. all groups). The HFD+ AICAR rats revealed a significant reduction in TNF-α to reach control levels; however, they still displayed a difference from the AICAR control group (*p* = 0.0483). This was accomplished with the low gene expression of SNARK observed in the AICAR groups (Figure 3A,B,D). Furthermore, AICAR + HFD rats exhibited significantly lower serum levels of IL-6 and IL1-β (Figure 5B,C; *p* < 0.0001 for HFD vs. all groups). These data verified that AICAR-mediated inhibition of the HGF/NF-κB pathway amended the HFD-induced inflammatory response. To assess if hepatic NAD^+^ levels were affected by HFD, we assayed hepatic NAD^+^ levels. Steatotic hepatic tissues exhibited a significant reduction in NAD^+^ levels compared to the controls. AICAR treatment restored NAD^+^ levels in the livers of the AICAR + HFD group (Figure 5D; *p* < 0.0001). Exerkines such as the myokine irisin, or the adipokine visfatin/NAMPT are signaling molecules released in response to exercise, with different roles in metabolic, neurologic, and cardiovascular diseases [13]. The HFD group revealed significantly higher extracellular visfatin concentration, which failed to enhance cellular NAD^+^ levels, compared to control groups. AICAR administration significantly abolished HFD-induced high extracellular visfatin levels (Figure 5E, *p* < 0.0001). In contrast, HFD-fed rats exhibited reduced serum irisin levels versus the controls, while HFD rats receiving AICAR had higher levels of serum irisin (Figure 5F; *p* < 0.0001). In order to assess hepatic oxidative stress, lipid peroxidation product (MDA) and plasma TAC were assayed. Marked oxidative stress was observed in HFD-fed rats as compared to their allied controls. The HFD group receiving AICAR exhibited a significant increase in TAC and lowered MDA levels (Figure 5G,H; *p* < 0.001), which confirmed the hepato-protective and antioxidant effects of AICAR.

### 2.6. AICAR Suppresses In Vitro Palmitate Induced Steatosis in HepG2 Cells Independently of AMPK

**Figure 6:**
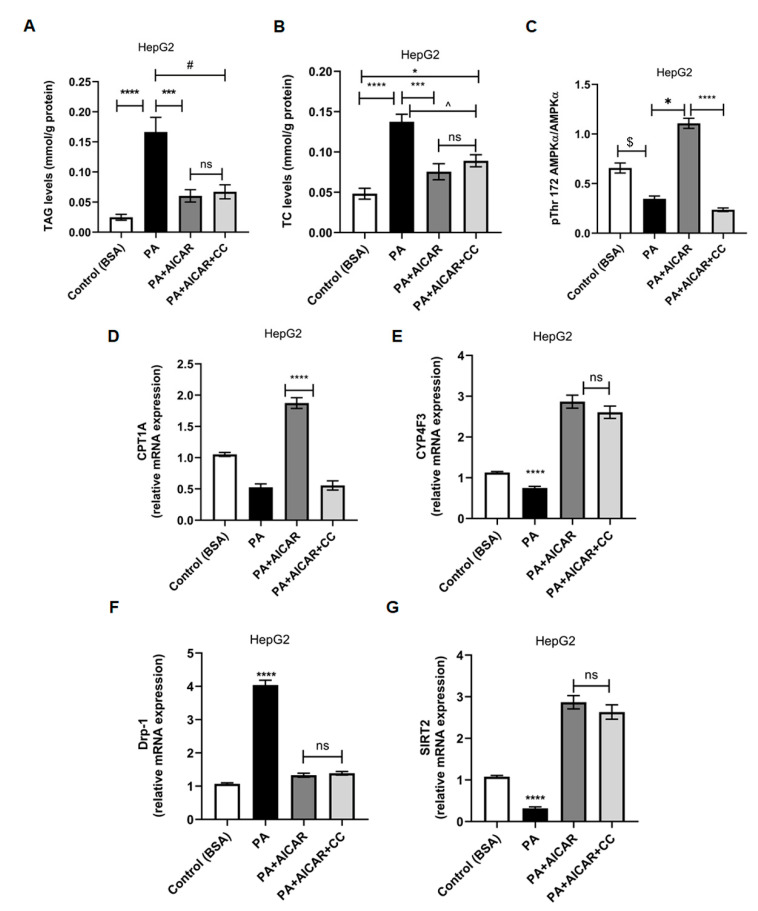
AICAR mitigates in vitro-induced steatosis and modulates *SIRT2*/*Drp1* expressions independently of AMPK. HepG2 cells were seeded at 3 × 10^5^ density in 6 multi-well plates in duplicates, then serum starved in 0.5% FBS containing medium for 24 h, and then exposed to 500 μM PA in culture medium containing 1% BSA (with or without 1 mM AICAR or 10 μM CC) for 24 h. Equal amounts of BSA were added to all control cells. (**A**) Mean TAG concentrations in HepG2 under different treatments; (****) *p* < 0.0001, (***) *p* = 0.0003, (#) *p* = 0.0006. (**B**) Mean TC concentrations in HepG2 under different treatments; (****) *p* < 0.0001, (***) *p* = 0.0003, (^) *p* = 0.0034 (*), *p* = 0.0137). (**C**) AMPK activity by ELISA as pThr172AMPKα/AMPKα ratio; (****) *p* < 0.0001, (*) *p* = 0.0173, ($) *p* = 0.0043 by using Kruskal–Wallis test. Inter-group comparisons were by Dunn’s post-hoc test and/or Mann–Whitney test (*n* = 6). (**D**) Relative mRNA expression of *CPTA1* in HepG2 cells under different treatments; (****) *p* < 0.0001 for PA vs. all. (**E**) Relative mRNA expression of *CYP4F3*; (****) *p* < 0.0001 for PA vs. all. (**F**) Relative mRNA expression of *Drp1* in HepG2 cells; (****) *p* < 0.0001 for PA vs. all. (**G**) Relative mRNA expression of *SIRT2* in HepG2 cells; (****) *p* < 0.0001. Significance by one-way ANOVA followed by Tukey’s post-hoc test, and data are presented as mean ± SEM, *n* = 6. *p ≤* 0.05 is considered significant; n.s.: non-significant. (PA: Palmitic acid; CC: Compound C; TAG: triacylglycerol; TC: total cholesterol; *CPTA1*: carnitine palmitoyl transferase 1A; *CYP4F3*: cytochrome P450 family 4 subfamily F member 3).

The AMPK-independent impacts of AICAR have been described, making it distinctive from all other AMPK particular activators [24]. To support our in vivo findings, in vitro steatosis was induced in HepG2 cells by treating them with palmitate (PA) at 0.5 mM for 24 h [31]. Steatosis was confirmed by the accumulation of triacylglycerol (TAG) in PA-exposed cells compared to other cells (Figure 6A; *p* < 0.0001 vs. Control, *p* = 0.0003 vs. AICAR treated, *p* = 0.0006 vs. PA + AICAR + CC). Total cholesterol (TC) concentrations were significantly elevated in PA cells (Figure 6B; *p* < 0.0001 vs. control, *p* = 0.0003 vs. AICAR treated, *p* = 0.0034 vs. PA + AICAR + CC), while PA+ AICAR cells showed lower TAG and TC values. To assess whether AMPK activity was mandatory in AICAR-mediated suppression of lipogenesis, Compound C (CC) (a known inhibitor of AMPK) was used. AMPK inhibition was confirmed by the reduction of the phospho-threonine 172 AMPKα ratio to total AMPKα, measured by ELISA (Figure 6C; *p* < 0.0001 vs. AICAR group). PA-treated cells revealed lower levels of AMPK activity (Figure 6C; *p* = 0.0043 vs. control, *p* = 0.0173 vs. AICAR treated). Interestingly, AICAR decreased TAG levels when combined with CC treatment (0.06 ± 0.01 for PA + AICAR vs. 0.07 ± 0.012 for PA + AICAR + CC), yet the values under CC remained higher than with AICAR only; however, this difference was statistically insignificant. Additionally, AICAR lowered TC values (0.076 ± 0.001 for PA + AICAR vs. 0.08906 ± 0.01 for PA + AICAR + CC), though this was non-significant. Yet, TC values under AMPK inhibition were still higher compared to control cells (0.023 ± 0.01) (Figure 6B; *p* = 0.0137). Mitochondrial β-oxidation is an important metabolic pathway of fatty acids that is altered by steatosis [32]. The mRNA expression of the β-oxidation key enzyme carnitine palmitoyl transferase 1A (*CPTA1*) was downregulated in response to PA, while AICAR treatment upregulated *CPTA1* gene expression and improved β-oxidation; this was an effect that seemed to be AMPK-dependent and was abolished by Compound C (Figure 6D; *p* < 0.0001; PA + AICAR vs. PA and PA + AICAR + CC). This implies that a basal AMPK activity is still required for the AICAR-mediated impact on β-oxidation.

In order to investigate alternative pathway(s) of the observed effects of AICAR on lipogenesis, expression of the cytochrome P450 family 4 subfamily F member 3 (*CYP4F3*) gene was analyzed. *CYP4F3* encodes for omega-hydroxylase 2 in ω-oxidation of fatty acid as an AMPK-independent alternative pathway to remove fatty acid overload [33]. *CYP4F3* expression was downregulated by PA (*p* < 0.0001). AICAR significantly upregulated *CYP4F3* gene expression by 2.5-fold relative to the controls, an increase that was not abolished by Compound C (Figure 6E; *p* < 0.0001). Therefore, AICAR appears to control lipogenesis independent of AMPK by promoting the ω-oxidation pathway. AICAR has been reported to reduce mitochondrial ROS generation and fission by inhibiting Drp1 phosphorylation at serine 637 [34]. Our in vivo results revealed decreased hepatic *Drp1* expression in the HFD + AICAR group (Figure 4D, *p* < 0.0001). To further elucidate AMPK’s role in AICAR-mediated inhibition of *Drp1*, we checked the expression of *Drp1* under Compound C inhibition. Interestingly, AICAR significantly downregulated *Drp1* expression in PA-treated cells with and without CC (Figure 6F; *p* < 0.0001). SIRT2 restores ROS-induced mitochondrial dysfunction via inhibition of Drp1 phosphorylation and its activity [35]. AICAR upregulated *SIRT2* gene expression in PA cells treated with AICAR and/or Compound C, irrelevant of AMPK, by 2.6, and 2.7-fold, respectively (Figure 6G; *p* < 0.0001). In addition, in HepG2 cells, TAG positively correlated with *Drp1* expression and negatively correlated with *SIRT2* and *CYP4F3* expression in PA + AICAR-treated cells with and without CC. TAG negatively correlated with *CPT1A* expression only in the PA + AICAR group. All correlations were of statistical significance (Table 1; *p* < 0.05). Therefore, AICAR can inhibit in vitro steatosis through AMPK-dependent or -independent mechanisms, depending on the cellular context. This inhibition is achieved partly by promoting ω-oxidation and relieving mitochondrial dysfunction by increasing SIRT2 expression, which further inhibits Drp1 activity and the direct repressive effect on its mRNA level.

### 2.7. AICAR Abrogates Lipotoxic-Induced Endoplasmic Reticular Stress

**Figure 7:**
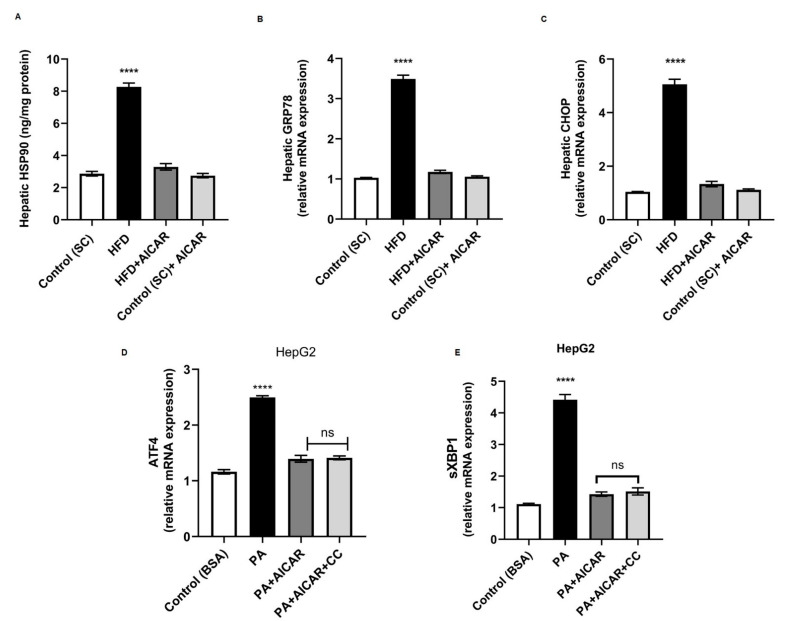
Impact of AICAR on lipotoxic-induced endoplasmic reticular stress. Male Wistar rats fed on HFD with or without AICAR for 8 weeks. (**A**) Mean hepatic Hsp90 levels measured by ELISA among all studied groups; (****) *p* < 0.0001 for HFD vs. all groups. (**B**) Hepatic *GRP78* relative mRNA expression in livers from all groups under study; (****) *p* < 0.0001 for HDF vs. all groups. (**C**) Hepatic *CHOP* relative mRNA expression in livers from all groups under study; (****) *p* < 0.0001 for HDF vs. all groups. HepG2 were treated as in (Figure 6) (**D**) Relative mRNA expression of *ATF4* in HepG2; *p* < 0.0001 for PA vs. all cell groups. (**E**) Relative mRNA expression of *sXBP1* in HepG2; (****) *p* < 0.0001 for PA vs. all cell groups. Significance by one-way ANOVA followed by Tukey’s post-hoc test, and data are presented as mean ± SEM; *n* = 15 for animal experiments, *n* = 6 for HepG2 cells. *p ≤* 0.05 is considered significant, n.s: non-significant. Hsp90: heat shock protein 90; *GRP78*: glucose regulated protein 78; *CHOP*: CCAAT-enhancer binding protein homologous protein; *ATF4*: activating transcription factor 4; *sXBP1*: spliced X-box-binding protein 1.

The Heat shock response (HSR) and ER stress have been described in the context of NAFLD pathogenesis [36]. To evaluate the changes in ER stress in the liver of HFD animals, the levels of heat shock protein 90 (Hsp90) and the key transcriptional markers of ER stress (glucose-regulated protein 78 [*GRP78*] and the gene of CCAAT-enhancer binding protein homologous protein [*CHOP*]) were assayed. The hepatic protein concentration of Hsp90 was increased in HFD-fed rats compared to others. The HFD + AICAR group revealed lower levels of hepatic Hsp90 levels compared to the HFD group (Figure 7A; *p* < 0.0001). Additionally, mRNA expression levels of *GRP78* and *CHOP* were both upregulated (3.4, and 4.8-fold, respectively, relative to control) in HFD-fed rats. The HFD + AICAR group showed reduced mRNA expressions of *GRP78* and *CHOP* by 2.9 and 3.9-fold compared to HFD-fed rats (Figure 7B,C; *p* < 0.0001). Although AICAR does not affect the ER stress response initiation, it does inhibit the major ER stress transcriptional effectors [37]. Consequently, we examined the effect of AICAR on the transcription of genes encoding for the ER effectors (spliced X-box-binding protein 1 [*sXBP1*] and activating transcription factor 4 [*ATF4*]) in HepG2 exposed to PA. PA + AICAR-treated cells exhibited a decline in mRNA expressions of *sXBP1* and *ATF4* by 3.1 and 1.8-fold, respectively (Figure 7D,E; *p* < 0.0001 for both). The AICAR-exerted downregulation was not reversed by AMPK inhibition by Compound C (Figure 7D,E; non-significant), indicating that the noted AICAR impact on relieving ER stress was AMPK-independent.

## 3. Discussion

**Figure 8:**
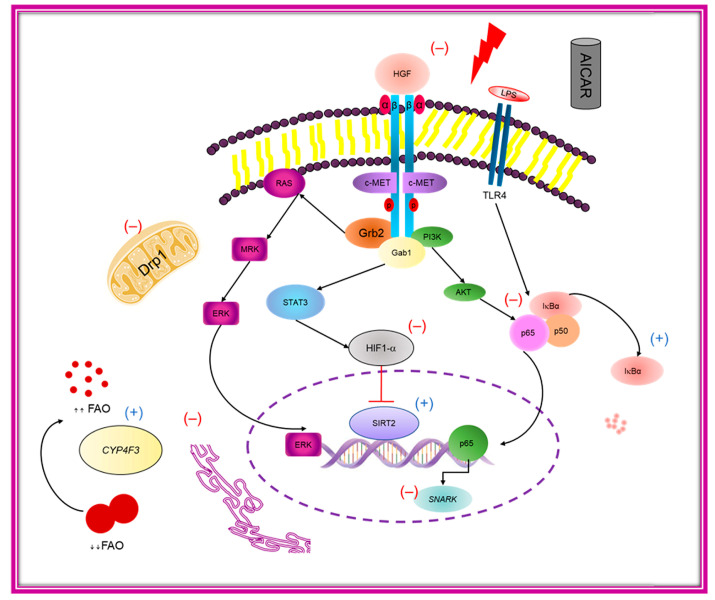
Proposed model of AICAR mechanism to mitigate NAFLD. AICAR treatment disrupts the HGF/c-Met signaling pathway with consequent blocking of the downstream effectors PI3K/AKT/IKK/NF-κB and STAT/HIF1-α. The anti-inflammatory action of AICAR is mediated via direct downregulation of genes under transcriptional control of NF-κB, particularly proinflammatory cytokines and SNARK. Inhibition of HIF1-α prevents *Drp1* expression that is also blocked by upregulated *SIRT2* expression. Aside from AMPK dominance, AICAR downregulates *Drp1*, *SNARK*, and ER stress response gene expression, which alleviates mitochondrial dysfunction and ER stress. Importantly, AICAR upregulates *CYP4F3* gene expression by inducing the alternative ω-pathway of fatty acid oxidation (Gab1:GRB2-associated-binding protein 1; Grb2: Growth factor receptor-bound protein 2; FAO: fatty acid oxidation).

Hepatic steatosis is a common finding in obesity, diabetes mellitus (DM) and metabolic syndrome (MS) with numerous complex biochemical, metabolic, and clinical manifestations. Several anti-diabetic drugs such as metformin and other AMPK activators have been widely used to treat NAFLD, yet their exact mechanism(s) of action in NAFLD remains largely obscure [38]. The old drug, AICAR, has drawn attention for its potential AMPK-independent actions at the cellular level [24]. The present work investigated novel mechanisms underlying AICAR mitigation of NAFLD-associated oxido-inflammatory damage by modulating HGF/NF-κB signaling, regulating ER stress and mitochondrial dysfunction, and restoring metabolic homeostasis regardless of AMPK dependence. In humans, circulating high levels of HGF positively correlate with insulin resistance and glycemic status and were reported to be elevated in obesity, hypertension, and metabolic syndrome [15,16]. This is in line with the current finding that HFD-fed rats exhibited higher serum levels of HGF compared to the HFD + AICAR group. Downstream analysis of the HGF pathway indicated augmented NF-κB activity in HFD-fed rats, which was prevented with concomitant AICAR administration, as evidenced by diminished p65 protein expression levels and upregulation of IκBα. This is well aligned with previous reports describing the role of NF-κB activity and IκBα degradation in the pathogenesis of NAFLD and the beneficial role of AICAR in downregulating the NF-κB pathway [39,40]. The noted increase in NF-κB activity is downstream of HGF that induces the NAFLD-associated transcriptome [18,19]. HGF/c-Met activates NF-κB in different cell types and contexts, including hepatic cells, via activation of the STAT3, PI3K/Akt, and ERK/MAPK signaling pathways (Figure 8) [19]. Numerous earlier studies revealed NF-κB activation by HGF signaling in hepatic cell lines. Moreover, inhibition of NF-κB blocked HGF-induced cellular proliferation [30]. Remarkably, AICAR inhibits NF-κB DNA binding in an AMPK-independent manner by preventing the recruitment of NF-κB and RNA polymerase II to target gene promoters; however, it does not induce direct p65 nuclear translocation [29]. Furthermore, a preclinical study revealed that Hsp90 inhibition facilitated HGF drug inhibition [41]. Importantly, our results showed decreased hepatic Hsp90 levels in the HFD + AICAR group. Additionally, metformin, a closely related drug, blocks the binding of c-MET and Gab1 and then inhibits Gab1 phosphorylation and activation. Therefore, we could propose a similar mechanism for AICAR. It is of note that there is an increase in HGF secretion by adipocytes among obese subjects due to TNF-α stimulation, increased adipocyte size, and posttranscriptional modification [42]. This is consistent with our finding that HFD-fed rats receiving AICAR had reduced TNF-α levels and adipocyte size. The observed adipocyte antiproliferative effects of AICAR were observed in AMPK loss-of-function studies and could be attributed to AICAR’s direct effects on the cell cycle [26]. NAFLD is associated with hypoxic stress due to altered lipid homeostasis and ROS generation. Importantly, HGF/c-MET signaling activates HIF1-α–mediated transcription that induces alterations in mitochondrial oxidative metabolism [43]. In the present study, AICAR reduced hepatic HIF1-α protein expression in HFD-fed rats. HIF-1α represses *SIRT2* expression and promotes obesity, while *SIRT2* knock-out animals develop obesity, hepatic steatosis, and ER stress [2,22]. This is relevant to our observed low SIRT2 expressions in in vivo and in vitro steatosis, which were upregulated by AICAR independent of AMPK. Intriguingly, a study has reported direct SIRT2 interaction and deacetylation of HIF-1α promoting its hydroxylation and ubiquitination [44]. This was enhanced by higher levels of cellular NAD^+^. Along this line, we observed a rise in hepatic NAD^+^ concentrations in the HFD + AICAR group. In the context of NAFLD pathogenesis, increased SIRT1 expression in response to AICAR treatment was AMPK mediated [45]; nevertheless, no study has explored AICAR’s impact on SIRT2.

Downstream analysis of the NF-κB pathway revealed increased SNARK expression and nuclear translocation in the livers of HFD-fed rats. SNARK is the only AMPK-related kinase under transcriptional control of NF-κB and in response to TNF-α stimulation. It is upregulated in response to a multitude of cellular stresses, human obesity, and HFD animal models [21].

In accordance with current findings, *SNARK* mRNA increases in myotubes exposed to palmitate or TNF-α [46]. Our demonstrated AICAR effects on SNARK expression were due to NF-κB inhibition and are kinase-independent due to the lack of SNARK enzymatic activation by AICAR [47]. The observed nuclear predominance of hepatic SNARK immunoreactivity is attributed to lipotoxic, oxidative, and ER stress, which drive its nuclear translocation [48]. SNARK activates hepatic yes-associated protein (YAP)/Hippo signaling, which plays a role in how NAFLD develops into NASH [49,50]. Interestingly, De Ran et al. [51] have reported AICAR-induced YAP inactivation. The mitochondrial biogenesis gene *PGC1α* stimulates hepatic fatty acid oxidation, reduces TAG, and is downregulated in obesity [21]. Herein, SNARK gene expression correlates negatively with that of *PGC1α* under AICAR administration. Queiroz et al. [21] reported *SNARK* transcription of murine *miR-696*, which binds and inhibits *PGC1α*. Based on our EM data, AICAR alleviated HFD-induced mitochondrial dysfunction, increased the number of mitochondria, promoted fusion, and decreased the fission process. This is explained by AICAR’s ability to modulate the SIRT2/PGC1α/Drp1 axis. SIRT2 modulates mitochondrial biogenesis to control the production of ROS levels by promoting PGC1α deacetylation [22]. Inhibition of mitochondrial fission suppresses hepatic oxidative stress as well as steatosis [9]. Moreover, AICAR-induced transcriptional downregulation of *Drp1* improved in vivo and in vitro steatosis. In agreement with our findings, Galloway et al. [7], in their *Drp1* transgenic mouse study, stated that *Drp1* depletion protected against steatosis and improved lipogenesis. Our findings also coincide with Choi et al. [9], who found that suppression of *Drp1* mRNA expression mitigated ethanol-induced steatosis in HepG2. Remarkably, our AICAR modulatory effects on mitochondrial dysfunction were AMPK-independent. SIRT2 inhibits Drp1 in a non-canonical way, where it promotes the deacetylation of MAPK kinase-1 (MEK1)/ERK or AKT1 with the subsequent inhibition of Drp1 phosphorylation [35]. Whether SIRT2 directly deacetylates Drp1 requires further investigation. In the current study, HFD-fed rats developed persistent weight gain, impaired glucose tolerance, insulin resistance, and increased HOMA-IR. NAFLD development was evidenced by histopathological findings. AICAR administration along with HFD significantly prevented weight gain and reduced hepatic steatosis, ballooning, and inflammation. Additionally, it improved dyslipidemia and reduced liver enzymes and triglycerides synthesis. This is in accordance with prior studies reporting improved hepatic steatosis by AICAR [38,52]. AMPK reportedly mediates AICAR’s beneficial metabolic actions; active Thr172 phospho-AMPK exhibits an anti-lipogenic effect by suppressing hepatic expression and activities of lipogenic enzymes and activates β oxidation [24]. This is consistent with our current findings where HFD + AICAR rats revealed reduced activities of the FAS and PAP lipogenic enzymes. Although we detected activation of AMPK in steatotic HepG2 cells treated with AICAR, AICAR can reduce TAG and to a lesser extent TC in cells treated with the AMPK-inhibitor Compound C. In this regard, studies on AMPK knock-out mice reveal that plasma fatty acids and triglyceride levels decrease independently of hepatic AMPK during AICAR administration. In addition, AMPK was reported to be non-essential for AICAR-induced fatty acid oxidation in skeletal muscles [53,54]. Our in vitro data demonstrated upregulation of the β-oxidation gene *CPT1A* in an AMPK-dependent way. This is consistent with preceding reports describing AICAR’s effect on *CPT1A* gene expression [32]. On the other hand, we proved that gene expression of *CYP4F3* was AMPK-independent. Thus, it is plausible that this *CYP4F3* upregulation is representative of increased ω-hydroxylation as an alternative pathway for removing the fatty acid overload by AICAR [33]. The biological significance of this route occurring in the liver and other organs is the generation of more soluble acids that are cleared by the kidneys, especially for those on low-carbohydrate or ketogenic diets. Despite the notion that ω-oxidation is catalyzed by enzymes that induce ROS and NAFLD progression [55], other studies consider it a rescue pathway for fatty acid oxidation disorders [56].

Along this line, Bumpus, et al. [57] described that AICAR upregulates the mRNA expressions of *CYP4F* murine homologs (*Cyp4a10*, *Cyp4a14*, *Cyp4a31*) independent of AMPK. *CYP4F3* catalyzes the inactivation of the proinflammatory leukotriene B4 by ω-oxidation in human neutrophils, which explains the anti-inflammatory effects of AICAR in HFD-induced steatosis. In the present in vitro study, *CYP4F3* expression was decreased by PA treatment and negatively correlated with TAG load; this could be attributed to its posttranscriptional modification and splicing [33,58]. In the context of NAFLD, gut Gram-negative bacteria and ROS induce toll-like receptor 4 (TLR4) stimulation. Furthermore, a heightened TLR4/NF-κB/TNF-α signaling pathway in hepatocytes and Kupffer cells release inflammatory cytokines and promote chronic inflammation [4,15]. Consistently, as observed herein, AICAR-ameliorated hepatic and systemic inflammatory response restored redox homeostasis. This is consistent with earlier reports describing the direct anti-inflammatory and antioxidant actions of AICAR [29,59]. In the present work, AICAR regulated circulating exerkines by restoring irisin and reducing circulating visfatin levels in HFD-fed rats. It is noteworthy that AICAR regulates a large part of the contracting skeletal muscle metabolome and proteome [26,60]. The AMPK-independent action of AICAR on fatty acid oxidation in skeletal muscle has been also reported [53]. Irisin is derived from its precursor, fibronectin type III domain-containing protein 5 (FNDC5), which is under expressed in obesity and NAFLD [15]. Similarly, AICAR restores irisin levels in HFD-fed rats. Recently, Guo and colleagues [61] reported that PA treatment decreased FNDC5 expression; this inhibition was alleviated by AICAR because it binds to a transcriptional repressor zinc finger protein ZFP57 and turns FNDC5 transcription on. Given that ZFP57 null cells failed to increase irisin levels in response to AICAR, it is intriguing to speculate that ZFP57 could be another AICAR-response-element binding protein, like zinc finger protein 692, that mediates the transcriptional activity of AICAR [62]. It should be noted that SIRT2 upregulation deacetylates and stabilizes irisin to abrogate NAFLD [63]. Visfatin/NAMPT exists in two forms: intracellular NAMPT (iNAMPT) that catalyzes NAD^+^ biosynthesis and modulates insulin sensitivity and lipid metabolism and extracellular eNAMPT/visfatin, an inflammatory cytokine that activates TLR4/NF-κB signaling in obesity and NAFLD [64]. Aligned with our work, chronic AICAR treatment was found to significantly increase PGC1α and iNAMPT expressions in rat skeletal muscles and in AMPK α null muscles [65,66]. Dysfunction of hepatocyte ER with subsequent alterations of folded proteins and lipid and calcium homeostasis is a hallmark of NAFLD pathogenesis. Mitochondrial dysfunction coupled with ER stress in NAFLD changes the hepatic transcriptome and secretome [11,12]. In this study, the expression of Hsp90, *GRP76*, and UPR downstream effectors (*ATF4*, *CHOP* and *sXBP1*) were downregulated in in vivo and in vitro steatosis in response to AICAR administration. Relevant to our data, higher circulating Hsp90 levels have been observed in obese NAFLD patients [67]. Moreover, independent from AMPK, AICAR interacts with and destabilizes Hsp90 in vivo [25]. GRP78 is a central upstream regulator of ER stress that activates stress sensors IRE1α, PERK, and ATF6 [2]. Activation of the PERK pathway causes overexpression of ATF4 and its downstream anti-apoptotic CHOP, which activates NF-κB signaling. The IRE1α pathway induces spliced sXBP1, which is connected to lipogenesis and NASH development [12]. In alignment with our data, higher gene expression levels of *GRP78*, *CHOP*, *ATF4*, and *sXBP1* have been associated with obesity, steatosis, and mitochondrial stress [68,69]. The mitigating effects of AICAR on ER stress have been well described as an AMPK activator [70,71]. The present work unravels the AMPK-independent actions of AICAR in alleviating PA-induced ER stress in HepG2 cells. AICAR significantly reduced the expression of the UPR downstream effectors *sXBP1* and *ATF4*. Consistent with this, it has been demonstrated that AICAR relieves ER stress response in human macrophages exposed to PA and in AMPK-α1 knock-down cells [37]. However, AICAR does not abolish ER stress response initiation; rather, it inhibits the mRNA and protein expression of all major transcriptional ER stress effectors [37]. Since AICAR’s specific actions are cell-specific and subjected to various factors, it is plausible that AICAR’s effects could be controlled by the expression of nucleoside transporter proteins, which influence intracellular ZMP accumulation and responses [72].

Further studies are warranted to specify the spatiotemporal localization of AICAR inside different cell types as well as pharmacological modification to overcome its poor oral bioavailability.

## 4. Materials and Methods

### 4.1. Chemicals

AICAR (PubChem CID: 17513), drug bank accession number: DB01700, Phosphatidic acid (PubChem CID: 446066), Palmitic acid (PubChem Substance CID: 24898107) and compound C (PubChem CID: 11524144) were purchased from Sigma-Aldrich (St. Louis, MO, USA). All other chemicals and solvents were of high analytical grade and were purchased from Sigma-Aldrich unless otherwise described.

### 4.2. Experimental Design and Animal Treatment Protocol

Sixty male Wistar rats weighing 120–150 g were purchased by the Faculty of Medicine, Tanta University. Animals were housed in sanitary well-ventilated polyplastic cages with steel wire tops (four per cage) and maintained under standard laboratory conditions of ambient temperature (25 ± 2 °C) and relative humidity (50 ± 5%) under a 12:12-h light dark cycle. Rats were fed standard rat chew and tap water ad libitum. The sample size was calculated based on a G* Power test (Version 3.1.9.7, Dusseldorf, Germany, http://www.gpower.hhu.de, accessed on 1 December 2022) [73]; the power: 0.95, the effect size (F): 0.2729762, and the alpha: 0.05.

Following acclimatization, the rats were randomly allocated into four groups of 15 rats each (Figure 1A). Group I (control [SC]) received rat standard chow (SC) for 8 weeks; group II (HFD) received HFD for 8 weeks; group III (HFD + AICAR) received HFD with a simultaneous intraperitoneal injection (i.p.) of AICAR at 0.7 g/kg body weight (b.w.) [74] every morning (from 8:00 to 10:00 A.M.) for 8 weeks in a single dose; group IV (Control [SC] + AICAR) received the same AICAR injection as group III for 8 weeks. AICAR was dissolved in 0.9% NaCl and heated at 37 °C for 20 min; all controls and HFD animals were injected with a corresponding volume of 0.9% NaCl every day. Standard chow (SC) for control groups was composed of 60% ground corn meal, 10% bran, 15% ground bean, 10% corn oil, 3% casein, and mineral and vitamins mix (1% each) that was obtained from (Melad Co, Aubor City, Cairo, Egypt). The high-fat diet (HFD) was composed mainly of 70% fat, 20% carbohydrates, and 10% protein. The meal comprised cooked caw fat, full cream milk, bread, and vegetables [27]. This study was conducted in accordance with the guiding principles of the National Institutes of Health for the care and use of laboratory animals (NIH Publications No. 8023, revised 1996) to minimize animal number and distress and approved by the Ethical Committee of Medical Research, Faculty of Medicine, Tanta University, Egypt (agreement code: 31778/09/17).

### 4.3. Blood and Tissue Collection

To determine whether NAFLD was successfully induced, a pilot study was performed before the start of the experimental period, and NAFLD was successfully confirmed by the histopathological examination of liver sections. At the end of the experimental period and after an overnight fast, animals were weighed and then anesthetized with an injection of pentobarbitone sodium (60 mg/kg i.p.). Blood samples were collected in test tubes with or without EDTA through cardiac puncture. The plasma samples and sera were separated by centrifugation (3500 rpm/min) for 10 min and were stored at −80 °C for further assays. Tissues were obtained after decapitation of anesthetized rats, rinsed in saline, weighed, snap frozen (20 mg hepatic tissues were snap frozen for NAD^+^ assay) and stored at −80 °C until analysis. For light and electron microscopy, tissues were fixed in 10% neutral-buffered formalin, 2.5% glutaraldehyde and/or 4% paraformaldehyde (PFA) and proceeded according to the specified protocols.

### 4.4. Liver Homogenates and Subcellular Fractionation

Liver pieces were homogenized in four volumes of ice-cold 0.25 M sucrose homogenization buffer containing (1 mM ethylene-diamine-tetra acetic acid, 0.2 mM dithiothreitol, 1% Triton X-100, 1 mM sodium orthovanadate and 10 mM Tris-HCl; pH 7.4) then centrifuged at 13,000 rpm for 15 min at 4 °C. Subcellular fractionation of nuclear, cytosolic, and microsomal fractions were performed as previously described [75,76]. For NF-κB assessment, a nucleocytoplasmic fractionation kit (Biovision Incorporated, Milpitas, CA, USA) was used according to the manufacturer’s instructions. All homogenates were then stored at −80 °C for future analysis.

### 4.5. Biochemical Assays

#### 4.5.1. Intraperitoneal Glucose Tolerance Test (IPGTT)

At the end of the eighth week, the rats received an intraperitoneal injection of two g/kg (b.w.) of glucose after fasting for 16 h. Blood samples were obtained from the tail vein, and blood glucose levels were monitored using the Bionime GM 100 glucometer (BIONIME Corporation, Dali, Taiwan) at 0, 15, 30, 60, 90 and 120 min right after injection of the glucose load. The ROC curve was constructed and the area under the ROC curve (AUC) was calculated.

#### 4.5.2. Lipid Profile, Redox and Glycemic Status

Fasting blood sugar (FBS) was measured by the oxidase method using a commercial kit (Biodiagnostic, Egypt). Total lipid profile, including total cholesterol (TC), triglycerides (TAG) and HDL-cholesterol (HDL-Ch), was measured by enzymatic-colorimetric methods (HUMAN, Wiesbaden, Germany) according to the manufacturer’s protocol. LDL cholesterol (LDL-Ch) concentration was calculated according to the Friedewald equation [77]. Detailed methods of biochemical and enzymatic assays are available online as Supplementary Materials and methods.

### 4.6. Western Blotting

Total protein extracts were prepared by homogenization of hepatic tissues on ice in lysis buffer containing 50 mM Tris-HCl pH 7.6, 250 mM NaCl, 1% Triton X-100, 0.5% Nonidet (P40), 3 mM EDTA, 3 mM EGTA, 10% glycerol, 2 mM DTT, 1 mM PMSF, 1 mM sodium orthovanadate and a protease inhibitor cocktail (Calbiochem, Merck, MA, USA), followed by brief sonication and then centrifugation at 13,000 rpm for 15 min at 4 °C. The supernatant was placed into a new tube, and the protein concentrations were assayed by a BCA protein assay kit (PierceTM, Rockford, IL, USA). Nuclear fractions and total proteins of 25 μg were used. Samples were boiled in 4× Laemmli sample buffer (Bio-Rad, Hercules, CA, USA) and separated on a 10% SDS PAGE (sodium dodecyl sulfate polyacrylamide gel), then electro-transferred onto polyvinylidene difluoride (PVDF) membranes (Merck, Millipore MA, USA). The PVDF membranes were blocked in 5% skimmed milk in 1× phosphate buffered saline and 0.1% Tween 20 (PBST) for 1 h at room temperature, followed by overnight incubation at 4 °C with primary antibodies (mouse anti-p65: SC-8008, mouse anti-IκBα: SC-1643, and mouse anti- Lamin B1: SC-374015) (loading control for nuclear fraction) (Santa Cruz Biotechnology, Inc., Santa Cruz, Santa Cruz Biotechnology, Inc., Texas, USA) at 1:1000 dilutions for all and rabbit anti-SIRT2 (ab211033, Abcam, MA, USA) at 1:1000 dilution. Mouse anti-β- actin antibody (#8226; Abcam, Cambridge, UK) at 1:5000 dilution was used as a loading control. After washing three times with PBST, blots were probed for 1 h at room temperature with secondary antibodies (anti-rabbit or anti-mouse IgG linked to horseradish peroxidase [ab6721 and ab205719, Abcam]) at 1:3000 dilution in PBST. After washing three times in PBST, protein bands were visualized using enhanced chemiluminescence by Immobilon™ reagent (Merck, Millipore, Darmstadt, Germany). Densiometric quantification of protein bands was determined by Fiji Image J software (Fiji 2.7.0, NIH, Bethesda, MD, USA) [78].

### 4.7. Histopathological and Electron Microscopy

Detailed methods are available online as Supplementary Materials and methods.

### 4.8. RNA Extraction, cDNA Synthesis, and Real Time PCR

Total RNAs from frozen hepatic tissues were isolated by a GeneJET RNA Purification Kit (#K0731, Thermo Fisher Scientific Inc., Waltham, MA, USA). For HepG2, 3 × 10^5^ cells were extracted by TRIzol^®^ Reagent (Invitrogen, Waltham, MA, USA) according to the manufacturer’s recommendations. RNA quantity and purity were measured at OD260 and OD260/280 ratios, respectively, using a NanoDrop spectrophotometer (NanoDrop Technologies, Inc. Wilmington, NC, USA). Total RNA was reverse transcribed to cDNA using the RevertAid H Minus Reverse Transcriptase Kit (#K1632, Thermo Fisher Scientific Inc.) according to the guidance of the manufacturer. RT-PCR reactions were accomplished using QuantiTect^®^ SYBR^®^ Green PCR (# 204143, Qiagen GmbH, Hilden, Germany) with respect to the manufacturer’s protocol. Target mRNA transcripts were quantified, relative to the housekeeping gene β- actin as endogenous control. Sequence-specific primers were synthesized by Integrated DNA Technologies (IDT, Coralville, IA, USA) and are depicted in Supplementary Table S1. The thermal profile for PCR was as follows: 95 °C for 15 min, followed by 40 cycles of 94 °C for 15 s, 60 °C for 30 s, and 72 °C for 30 s. All reactions were performed in duplicate. The data were automatically generated by StepOnePlus™ Real-Time PCR Systems (Applied Biosystems, Foster City, CA, USA) and presented as relative expression of the gene of interest relative to the internal control gene (β-actin) by 2 ^(−ΔΔCT)^ method [79].

### 4.9. Cell Culture and Treatment

Human hepatoma cell lines (HepG2 cell) were from the American Type Culture Collection (ATTCC, Manassas, VA, USA). Cells were cultured in 75 cm^2^ tissue culture flasks (CELLSTAR, Greiner Bio-One International GmbH, Austria) at 80% confluency in Dulbecco’s Modified Eagle’s Medium (DMEM) (UFC Biotech, Riyadh, Saudi Arabia) supplemented with 10% fetal bovine serum (Invitrogen, Waltham, MA, USA), 100 units/mL penicillin, and 100 mg/mL streptomycin (Invitrogen). Cells were incubated at 37 °C in a humidified atmosphere containing 5% CO2. Palmitate acid (PA) stock solution was prepared as previously reported [80,81]. Fatty acid-free bovine serum albumin (BSA) was used as a vehicle for PA treatment. Compound C (CC) and AICAR were dissolved in dimethyl sulfoxide (DMSO) and were added to the culture medium at final concentrations of 10 μM and 1 mM, respectively. The final concentration of DMSO was <0.1% and did not affect cell viability. HepG2 cells were seeded in 6 well plates in triplicates. After reaching 70% confluence, cells were serum starved (0.5% FBS) and exposed to 500 μM PA in a culture medium containing 1% BSA, with or without AICAR or CC, for 24 h. Equal amounts of (BSA) were added to all control cells. Cells were divided into four groups: control (BSA only control); steatosis (PA only); AICAR-treated (PA + AICAR) and AICAR and CC treated (PA + AICAR + CC), as specified in figure legends. Cell viability was assessed by trypan blue, and steatosis induction was confirmed by measuring intracellular lipids and proteins.

### 4.10. Measurement of Intracellular Lipid Levels

Levels of triacyglycerols (TAG) and total cholesterol (TC) in HepG2 cells were quantified by enzymatic colorimetric assay (BioVision, Milpitas, CA, USA) according to the manufacturer’s instructions. Results were expressed relative to total proteins that were assayed by a BCA protein assay kit.

### 4.11. Statistical Analysis

Statistical analysis was performed using Graph Pad Prism 8.0.2 software (GraphPad Inc., San Diego, CA, USA). Results were presented as mean ± s.e.m. (standard error of the mean). Multiple comparisons were performed by one-way analysis of variance (ANOVA) followed by Tukey’s post-hoc test. Nonparametric data were analyzed using the Kruskal–Wallis test, followed by Dunn’s post-hoc test. Comparisons between the two groups were performed by the Mann–Whitney U-test. Correlations were analyzed using or Pearson’s correlation coefficients. Statistical significance was considered when the *p*-value was ≤0.05.

## 5. Conclusions

The salient finding of this study is the observed AMPK-independent actions of AICAR in the prevention of hepatic steatosis; these AICAR actions combat oxidative stress and inflammation and enhance mitochondrial structure and function. As depicted in Figure 8, the elaborated work in this study provides a mechanistic insight into AICAR’s ameliorative effects in NAFLD through disrupting the HGF/NF-κB/SNARK signaling pathway, abrogating mitochondrial dysfunction via Drp1/SIRT2 axis, and relieving ER stress. Taken together, its impact on normalizing cytokine and exerkine levels and the ROS and inflammatory response may represent a prophylactic and therapeutic approach to preventing NASH and hepatocellular carcinoma development, particularly by inhibiting SNARK oncogene among a high-risk population. Interfering with the HGF/NF-κB/SNARK pathway may represent a future prophylactic and therapeutic approach for NAFLD in humans.

## Figures and Tables

**Table 1:** Relationship of Triglyceride Contents (mmol/gm protein) to *SIRT2*, *Drp1*, *CPT1A*, *CYP4F3* mRNA Expression in HepG2 Treated with AICAR.

	BSA (Control)		PA		PA + AICAR		PA + AICAR + CC	
	*r*	*p*	*r*	*p*	*r*	*p*	*r*	*p*
***SIRT2***	**−0.8464**	**0.0336**	**−0.9062**	**0.0128**	**−0.8897**	**0.0176**	**−0.9605**	**0.0023**
***Drp1***	−0.3030	0.5594	**0.9114**	**0.0114**	**0.9182**	**0.0098**	**0.9953**	**<0.0001**
***CPT1A***	0.7872	0.0631	−0.5717	0.2359	**−0.9959**	**<0.0001**	−0.2146	0.683
***CYP4F3***	−0.09681	0.8552	**−0.9625**	**0.0021**	**−0.8897**	**0.0176**	**−0.8971**	**0.0153**

HepG2 cells were treated with Palmitate (PA) at 500 μM with or without AICAR (1 mM) or Compound C 10 μM (C) or BSA (control). Values are Pearson correlation coefficients. Bold font marks significance at *p* < 0.05. (*SIRT2*: Sirtuin 2; *Drp1*: Drp1: Dynamin-related protein1; *CPT1A*: Carnitine palmitoyl transferase I isoform A; *CYP4F3*: cytochrome P450 family 4 subfamily F member 3 (Gene coding for the enzyme Leukotriene-B4 omega-hydroxylase 2).

## Data Availability

Data available on request.

## Supplementary Materials

The following supporting information can be downloaded at: https://www.mdpi.com/article/10.3390/ijms24043367/s1. References [82,83,84,85,86,87,88,89,90,91,92,93] are cited in the supplementary materials.
