# Supplementary material for: AICAR Ameliorates Non-Alcoholic Fatty Liver Disease via Modulation of the HGF/NF-κB/SNARK Signaling Pathway and Restores Mitochondrial and Endoplasmic Reticular Impairments in High-Fat Diet-Fed Rats
Source: Int J Mol Sci. 2023 Feb 8;24(4):3367. doi: 10.3390/ijms24043367 (PMC9959470; doi:10.3390/ijms24043367)
Supplement: Supplementary file 1 [file ijms-24-03367-s001.zip › ijms-2140038-supplementary/Supplementary data/Supplementary Figure S1 Legend.pdf]

Supplementary Figure S1 Legend

Higher magnification photomicrographs of transmission electron microscope of hepatic sections from HFD-fed and HFD+AICAR rats. HFD-fed group showed enlarged swollen fragmented mitochondria (M), (*) cristolysis, swollen rough endoplasmic reticulum (RER) (arrow), lipid droplets (ld) and rarefied cytoplasm.

HFD+AICAR group showed normal size shaped tubular mitochondria (M), cristae, double head arrows point at fusion process and normal RER (arrow). Scale bar 500nm
