# Supplementary material for: AICAR Ameliorates Non-Alcoholic Fatty Liver Disease via Modulation of the HGF/NF-κB/SNARK Signaling Pathway and Restores Mitochondrial and Endoplasmic Reticular Impairments in High-Fat Diet-Fed Rats
Source: Int J Mol Sci. 2023 Feb 8;24(4):3367. doi: 10.3390/ijms24043367 (PMC9959470; doi:10.3390/ijms24043367)
Supplement: Supplementary file 1 [file ijms-24-03367-s001.zip › ijms-2140038-supplementary/Supplementary data/Supplementary methods.pdf]

Supplementary Materials and Methods

1. *Biochemical Assays*

1.1 Assessment Of Oxidant/Antioxidant Status in Liver Tissues

Determination of liver malondialdehyde (MDA) level was according to [1]. Plasma total antioxidant capacity (TAC) was assayed by enzymatic colorimetric method using commercial kit (Biodiagnostic, Egypt) [2].

1.2 Hepatic NAD⁺ Assay

NAD^+ levels in hepatic tissues were assayed by colorimetric method using commercially available kit according to manufacturer's instructions (EnzyChromTM #E2ND002, BioAssay Systems, Hayward, CA, USA,). Twenty mg hepatic tissues were used and homogenized in NAD^+ assay buffer. Optical density was measured at 0 and 15 minutes in a 96-well plate at 565 nm by using a microplate reader (BioTek, ELx808). NAD^+ concentrations were calculated using the following formula: NAD^+ = ΔΔ Sample - ΔΔ Blank/slope*n (where n is the dilution factor). Values were normalized to protein content.

1.3 Enzymes Assays

Phosphatidate phosphohydrolase (PAP) was measured by a method of [3] with slight modification. The enzyme was assayed at 37°C in a buffer containing 50 mmol/L Tris-HCl (pH 7.4), 1 mmol/L of phosphatidate, 1.25 mmol/L MgCl, and liver microsomes (50 μg of protein). The reaction was stopped by the addition of a solution containing (0.13% sodium dodecyl sulfate, 1.25% ascorbic acid, 0.32% ammonium molybdate-4H_2O, and 380 mmol/L H_2SO_4. The Color was developed at 45°C for 20 min at 820 nm. Non-enzymatic phosphate release was determined by inactivating the enzymes through boiling for 1 min without substrate. Pap activity was expressed as the amount of enzyme which catalyzes the release of 1 μmole of phosphate per min under the standard conditions as nanomoles per milligram of protein in one minute. Protein concentration was measured by Bradford assay with bovine serum albumin as a standard [4]. Serum activities of aspartate aminotransferase (AST) and alanine aminotransferase (ALT) activities were assayed by commercially available kits (HUMAN, Wiesbaden, Germany) according to supplier's instructions. The enzyme activity of fatty acid synthase (FAS; EC2.3.1.85) was performed as previously reported [5].

2. **Enzyme-linked Immunosorbent Assays**

Circulating blood levels of HGF, Visfatin, Irisin and insulin were immunoassayed using commercial ELISA kits (# MHG00, Quantikine®, R&D, MN, USA; #EIA-VIS-1, RayBiotech, Inc., Norcross, GA, USA; # 201-11-1713, Sunred Biological Technology Co., Ltd. Shanghai, China; #ERINS; Invitrogen, Waltham, MA, USA) respectively according to the manufacturer's guidelines. Serum inflammatory cytokines were assayed by ELISA; Tumor necrosis factor alpha (TNFα), Interleukin 6 (IL-6) and Interleukin 1-β (IL1-β) were from (#BMS622, #BMS625 and #BMS630; Thermo Fisher Scientific Inc., Waltham, MA, USA). AMPK activity was calculated by active/ total AMPK ratio. Threonine 172 phospho-AMPKα and total AMPKα were assayed using human sandwich PathScan ELISA Kits (# 7959C and #7961C, Cell Signaling, Danvers, MA, USA) respectively according to the manufacturer's guidance. Nuclear levels of hepatic NF-κB were assayed by sandwich ELISA kit (# SEB824Ra, Cloud Clone Corp., Katy, TX, USA), with assay range (0.312-20 ng/ml); sensitivity: 0.114ng/ml. Levels of Heat shock protein 90 (Hsp90) were detected by sandwich ELISA kit (# SEA823Ra, Cloud Clone Corp.,), with assay range (0.156-10ng/ml); sensitivity: 0.055 ng/ml. All ELISA assays with performed according to maker's protocols. All plates were assayed at 450 nm using a microplate reader (BioTek, ELx808, Agilent, Winooski, VT, USA) and data analyzed by Gen5 (Version 2.07, BioTek). The homeostasis model assessment index (HOMAIR) was calculated using the formula of fasting glucose (mmol/L) × fasting insulin (μIU/mL)/ 22.5 [6].

3. *Histopathological Examination of Liver*

liver tissues were fixed in 10% neutral-buffered formalin for 24 hours were dehydrated and embedded in paraffin. 5μm thick sections were stained with Hematoxylin and Eosin [7] for histological examination by light microscopy (Leica Imaging System LTD., Cambridge, UK). Histological scoring

of NAFLD activity were semi-quantitatively performed according to [8] where steatosis; (0–3), lobular inflammation; (0–2), hepatocellular ballooning; (0–2) and N.D., not detected.

4. *Immunohistochemistry of Liver*

For immunohistochemistry, tissues fixed in 4% PFA, were embedded in paraffin and 4μm thick sections were then deparaffinized, rehydrated and subjected to antigenic retrieval with sodium citrate. Tissue sections were blocked in 5% normal goat serum in PBST for one hour then incubated with primary anti-SNARK antibody (# PA5-102344, Invitrogen, Thermo Fisher Scientific, Inc., Waltham, MA, USA) 1:100 dilution at 4°C, overnight in a humidified chamber. Immunoperoxidase secondary detection system with secondary antibody was used (# DAB150, Millipore, Merck, Darmstadt, Germany) according to the manufacturer's protocol which used 3,3-diaminobenzidine (DAB) as chromogen. Immunoreactivity of SNARK appeared as brown staining of variable intensities. Slides were viewed by Olympus BX51 (Olympus Optical Co., LTD., Tokyo, Japan) with a digital camera (Olympus DP50; Olympus Optical Co.) and image were acquired by (Soft Imaging System GmbH, Münster, Germany). Quantifications of pixel intensities of hepatic SNARK immunoreactivity were performed in Fiji as previously described [9].

5. *Electron microscopy and mitochondrial morphology*

For electron microscopy (EM) tissues were fixed in 2.5% glutaraldehyde buffer and then in osmium tetroxide. Specimens were washed and dehydrated in alcohol. After that they were inserted in epoxy resin to prepare blocks for ultra-thin sections, that were subjected to staining by uranyl acetate and lead citrate. EM was performed by a transmission electron microscope (JEOL, Tokyo, Japan) at the electron microscopy unit, Tanta University. Morphometric analysis was carried out to assess mitochondrial morphology on 80kV electron micrographs according to [10] using Fiji. Mitochondrial morphology was assisted by two indices: aspect ratio (AR) and form factor (FF) values. AR is length-to-width ratio and FF equals (Pm2/4 Am), where Pm is the perimeter and Am is the area of mitochondrion. AR value of 1 means a circle, while higher values indicate elongated mitochondria. FF value of 1 signifies non-branched mitochondrion and higher FF one indicates a longer branched mitochondrion. A decrease in aspect ratio and/or form factor indicates mitochondrial fragmentation [11, 12]. Five measurements were taken for each ultrathin section of each group.

References

1. Ohkawa, H.; Ohishi, N.; Yagi, K., Assay for lipid peroxides in animal tissues by thiobarbituric acid reaction. *Anal Biochem* **1979,** 95, (2), 351-8.
2. Koracevic, D.; Koracevic, G.; Djordjevic, V.; Andrejevic, S.; Cosic, V., Method for the measurement of antioxidant activity in human fluids. *J Clin Pathol* **2001,** 54, (5), 356-61.
3. Yanagita, T.; Han, S. Y.; Wang, Y. M.; Tsuruta, Y.; Anno, T., Cycloalliin, a cyclic sulfur imino acid, reduces serum triacylglycerol in rats. *Nutrition* **2003,** 19, (2), 140-3.
4. Bradford, M. M., A rapid and sensitive method for the quantitation of microgram quantities of protein utilizing the principle of protein-dye binding. *Anal Biochem* **1976,** 72, 248-54.
5. Keshk, W. A.; Zineldeen, D. H.; Wasfy, R. E.; El-Khadrawy, O. H., Fatty acid synthase/oxidized low-density lipoprotein as metabolic oncogenes linking obesity to colon cancer via NF-kappa B in Egyptians. *Med Oncol* **2014,** 31, (10), 192.
6. Matthews, D. R.; Hosker, J. P.; Rudenski, A. S.; Naylor, B. A.; Treacher, D. F.; Turner, R. C., Homeostasis model assessment: insulin resistance and beta-cell function from fasting plasma glucose and insulin concentrations in man. *Diabetologia* **1985,** 28, (7), 412-9.
7. Suvarna, K. S.; Layton, C.; Bancroft, J. D., *Bancroft's Theory and Practice of Histological Techniques E-Book*. Elsevier Health Sciences: 2018.
8. Kleiner, D. E.; Brunt, E. M.; Van Natta, M.; Behling, C.; Contos, M. J.; Cummings, O. W.; Ferrell, L. D.; Liu, Y. C.; Torbenson, M. S.; Unalp-Arida, A.; Yeh, M.; McCullough, A. J.; Sanyal, A. J.; Network, N. S. C. R., Design and validation of a histological scoring system for nonalcoholic fatty liver disease. *Hepatology* **2005,** 41, (6), 1313-21.
9. zineldeen Curcumin attenuates cytoplasmic/endoplasmic reticulum stress, apoptosis and cholinergic dysfunction in diabetic rat hippocampus.

10. Merrill R.A., F. K. H., Strack S, *Techniques to Investigate Mitochondrial Function in Neurons*. Humana Press: New York, NY. , 2017; Vol. 123.
11. Ryan, Z. C.; Craig, T. A.; Folmes, C. D.; Wang, X.; Lanza, I. R.; Schaible, N. S.; Salisbury, J. L.; Nair, K. S.; Terzic, A.; Sieck, G. C.; Kumar, R., 1α,25-Dihydroxyvitamin D3 Regulates Mitochondrial Oxygen Consumption and Dynamics in Human Skeletal Muscle Cells. *J Biol Chem* **2016,** 291, (3), 1514-28.
12. Koopman, W. J.; Distelmaier, F.; Esseling, J. J.; Smeitink, J. A.; Willems, P. H., Computer-assisted live cell analysis of mitochondrial membrane potential, morphology and calcium handling. *Methods (San Diego, Calif.)* **2008,** 46, (4), 304-11.
