# Supplementary material for: AICAR Ameliorates Non-Alcoholic Fatty Liver Disease via Modulation of the HGF/NF-κB/SNARK Signaling Pathway and Restores Mitochondrial and Endoplasmic Reticular Impairments in High-Fat Diet-Fed Rats
Source: Int J Mol Sci. 2023 Feb 8;24(4):3367. doi: 10.3390/ijms24043367 (PMC9959470; doi:10.3390/ijms24043367)
Supplement: Supplementary file 1 [file ijms-24-03367-s001.zip › ijms-2140038-supplementary/Supplementary data/Supplementary Online Materials cover.pdf]

Supplementary Online Materials

AICAR Ameliorates Non-alcoholic Fatty Liver Disease via Modulation of the HGF/ NF-κB/ SNARK Signaling Pathway and Restores Mitochondrial and Endoplasmic Reticular Impairments in High-Fat Diet-Fed Rats

Doaa Hussein Zineldeen [1,2*] MD, PhD, Nahid Mohamed Tahoon [3] , MD, PhD and Naglaa Ibrahim Sarhan [4] ,MD, PhD

[1] Medical Biochemistry and Molecular Biology Department, Faculty of Medicine, Tanta University, Tanta 6632110, Egypt
[2] College of Medicine, Sulaiman AlRajhi University, Albukairiyah 51942, Saudi Arabia
[3] Physiology Department, Faculty of Medicine, Tanta University, Tanta 6632110, Egypt; nahidtahoon@med.tanta.edu.eg
[4] Histology Department, Faculty of Medicine, Tanta University, Tanta 6632110, Egypt; naglaa.sarhan@med.tanta.edu.eg

* Correspondence:
Doaa Hussein Zineldeen, MD, PhD, Nagoya, Japan.
Professor of Medical Biochemistry and Molecular Biology
Address: Department of Medical Biochemistry and Molecular Biology, Faculty of Medicine, Tanta University, El-Geish Street, Tanta, EL-Gharbia, Egypt. Postal No: 31527
E-mail: zineldeen@gmail.com, d.zineldeen@sr.edu.sa
Tel: +20403337544
Fax Number: +20403302785

Commented [MDPI1]: We added postal codes in all affiliations, please confirm

Commented [MDPI2R1]: I confirm
