# Supplementary material for: AICAR Ameliorates Non-Alcoholic Fatty Liver Disease via Modulation of the HGF/NF-κB/SNARK Signaling Pathway and Restores Mitochondrial and Endoplasmic Reticular Impairments in High-Fat Diet-Fed Rats
Source: Int J Mol Sci. 2023 Feb 8;24(4):3367. doi: 10.3390/ijms24043367 (PMC9959470; doi:10.3390/ijms24043367)
Supplement: Supplementary file 1 [file ijms-24-03367-s001.zip › ijms-2140038-supplementary/Supplementary data/Supplementary Table S1.pdf]

Supplementary Table S1: The primer sequences used in this study

Gene name	Primer sequence	Accession number	Amplicon
Rat *Drp1*	Forward: 5′- ATTGAAGGAACGGCAAAGTACATT -3′<br>Reverse: 5′- CAGATTCTAAGGTTCGCCCAAA -3′	NM_053655.3	100 bp
Rat *SNARK*	Forward: 5′- CTGTACATCCTGGTGCATGG -3′<br>Reverse: 5′- ACGTAACCAGTCCGCCATA -3′	NM_001007617.1	294 bp
RAT *GRP78*	Forward: 5′- GTTCTGCTTGATGTGTGTCC -3′<br>Reverse: 5′- TTTGGTCATTGGTGATGGTG -3′	NM_013083.2	350
Rat *CHOP*	Forward: 5′-GGAGAAGGAGCAGGAGAATG -3′<br>Reverse: 5′- GAGACAGACAGGAGGTGATG -3′	NM_001109986.1	176
Rat *β-Actin*	Forward: 5′- CAGGGTGTGATGGTGGGTATGG-3′<br>Reverse: 5′- AGTTGGTGACAATGCCGTGTTC-3′	NM_031144.3	115
Human *Drp1*	Forward: 5′-CACTTGTGGATTTGCCAGGAATGACC -3′<br>Reverse: 5′- TGCGACCATCTGGATCTACCTCTCTT-3′	NM_001278464.2	198 bp
Human SIRT2	Forward: 5′- CATCTCTAACTGCCCCCACG -3′<br>Reverse: 5′- TTTACTTAGCCACAGGCCCC -3′	NM_001193286.2	116 bp
Human CPTA1	Forward: 5′- CCTCCAGTTGGCTTATCGTG -3′<br>Reverse: 5′- TTCTTCGTCTGGCTGGACAT -3′	NM_001031847.3	133 bp
Human CYP4F3	Forward: 5′- GAGGAGGTTGTGTGGGACAAGG -3′<br>Reverse: 5′- GTGGAAGATGCGGACGATTGCG -3′	NM_000896.3	346 bp
Human *ATF4*	Forward: 5′- CTCCGGGACAGATTGGATGTT -3′<br>Reverse: 5′- GGCTGCTTATTAGTCTCCTGGAC -3′	NM_001675.4	165 bp
Human *sXBP1*	Forward: 5′- TTCCTTACCAGCCTCCCTTT -3′<br>Reverse: 5′- AGGTGCTTC CTCGATTTTCA -3′	NM_001079539.2	169 bp
Human *β-actin*	Forward: 5′- CCAACCGCGAGAAGATGA -3′<br>Reverse: 5′- CCAGAGGCGTACAGGGATAG -3′	NM_001101.5	97 bp
